# Applying the Adjusted Chinese Dietary Balance Index-16 to Assess the Dietary Quality of Chinese Postpartum Lactating Mothers

**DOI:** 10.3390/nu15214499

**Published:** 2023-10-24

**Authors:** Junyue Jiang, Jiating Huang, Yanyan Su, Yu Wang

**Affiliations:** School of Public Health, Lanzhou University, Lanzhou 730033, China; jiangjy21@lzu.edu.cn (J.J.); huangjt21@lzu.edu.cn (J.H.); suyy19@lzu.edu.cn (Y.S.)

**Keywords:** Chinese Dietary Balance Index-16, dietary quality, lactating women

## Abstract

A balanced diet is considered necessary in maternal recovery and neonatal development; however, the dietary quality of lactating mothers in China has not been systematically evaluated in different regions and stages of lactation. In addition, the release of the Chinese Dietary Guidelines in 2022 implies that the dietary index method needs to be adjusted accordingly. In this study, the adjusted Chinese Dietary Balance Index-16 (DBI-16) was used to assess the dietary quality of lactating women, referred to as the Dietary Balance Index for lactating women (DBI-L). This study is part of the MUAI study, in which dietary intake and demographic characteristics of lactating mothers from six cities in China and at different stages of lactation were obtained through a self-administered questionnaire and a food frequency questionnaire; 2532 puerperal women were included. According to the DBI-L, 66.2% of participants had inadequate dietary intake (79.1% vegetables, 79.1% fruits, 86.7% dairy products, 39.7% soybeans, and 69.4% fish products, respectively), 57.8% had excessive intake (76.0% cereals, 64.4% meat, and 29.1% eggs, respectively) and 92.2% had unbalanced dietary consumption. Dietary quality was optimal for mothers in the first month after delivery, and the dietary quality of mothers in economically developed places such as Shanghai and Guangzhou was significantly better than that in less developed places such as Lanzhou and Changchun. The dietary quality of lactating women in China is imbalanced, with excessive and inadequate dietary intake. The country should strengthen nutritional interventions for lactating mothers, especially in economically underdeveloped regions.

## 1. Introduction

Dietary nutrition of lactating mothers directly affects the growth and development of newborns. Also, it serves the function of repairing damage to the maternal body caused by delivery and promoting the recovery of maternal bodily functions [1]. Improper diet of lactating mothers during the puerperium not only leads to reduced milk production, which directly affects the nutrient quality of milk, but also exposes both the lactating mother and the developing infant to a higher risk of disease, mental disorders, and death [2,3]. In recent years, the Chinese Nutrition Society (CNS) has been revising the dietary guidelines (2022) for Chinese residents, which provide more scientific and balanced dietary recommendations for lactating women [4]. However, Chinese puerperal women are influenced by the traditional Chinese custom “Zuo Yue Zi”, which means the mother will be treated with a special diet and restricted activities for one month or more after delivery, which is different from the dietary patterns of other regions and countries [5]. During the puerperium, women usually consume carbohydrates and meats such as eggs, meat, animal offal, rib soup, fish soup, congee, and noodles to help replenish blood and increase breast milk production; cold and raw foods, such as vegetables and fruits, seafood, and condiments such as salt are limited [6,7]. In addition, the geographical area of China is vast, and the dietary habits of different regions vary considerably. Considering the complex factors influencing maternal diet in China, it is essential to investigate and assess the dietary nutritional status of Chinese mothers according to current dietary guidelines.

Dietary guidelines refer to the guidance issued by nutritional health authorities for the general public of a region or country, based on nutritional principles and combined with the actual situation a the local or national context, intending to promote rational nutrition to improve health and educate nationals on how to make wise and feasible food choices and adjust their dietary patterns [4]. Each country will formulate its dietary guidelines according to its dietary habits and characteristics, as well as the current health problems it is facing, to better guide its citizens toward a healthy diet and reduce the occurrence of chronic diseases.

The Dietary Guideline for Chinese Residents was first published in 1989 and revised three times in 1997, 2007, and 2016. The latest version of the guidelines will be published and implemented in 2022. With the development of the times, the dietary and nutritional structure of Chinese residents and the incidence of chronic diseases have changed significantly, so it is imperative to revise the new dietary guidelines promptly based on the social conditions at the time. The revised Dietary Guidelines for Chinese Residents 2022 are closer to reality and easier to follow in real life. Firstly, it is proposed for the first time that the dietary pattern along the southeast coast—rich in vegetables and fruits, with frequent consumption of fish, shrimp, and other aquatic products, soybean products and dairy products, and light cooking with less salt—represents the “Oriental Healthy Dietary Pattern” of China. Secondly, the “1 + 9” model has refined dietary guidelines for specific groups of people, including dietary guidelines for the general public aged 2 years and above, as well as the guidelines for nine specific groups of people. These nine groups are women preparing for and during pregnancy, breastfeeding women, infants from 0 to 6 months of age, infants and toddlers from 7 to 24 months of age, preschoolers, school-age children, elderly people in general, elderly people in advanced age, and vegetarian people. Lastly, “sharing meals with chopsticks and spoons” was emphasized. The COVID-19 epidemic reminds us to pay attention to public and personal hygiene and to promote healthy and civilized lifestyles. Adhering to hygienic measures, such as sharing chopsticks and spoons, and sharing meals or portions to avoid the occurrence and spread of foodborne illnesses is of great significance in safeguarding public health. For lactating women, the 2022 edition of the Dietary Guidelines has revised five core recommendations, including food variety but not overdose during the puerperium, balanced nutrition during lactation, increased intake of high-quality proteins and vitamin A, and moderate exercise. In addition, the 2022 edition of the balanced dietary pagoda for lactating women has revised the recommended intake levels of iodized salt, oil, fish, poultry, eggs, fruits, cereals, and other food groups, and the DBI-L scoring rules will also be revised accordingly based on these revisions.

Here, we compare the Chinese and U.S. Dietary Guidelines to emphasize the appropriateness of designating dietary guidelines based on national dietary patterns and establishing a dietary balance index [8]. The U.S. published the first version of the U.S. Dietary Goals in 1977 and renamed them the U.S. Dietary Guidelines in 1980, reflecting the practicality of the change from “goals” to “guidelines”. On 9 December 2020, the U.S. Department of Agriculture (USDA) and the Department of Health and Human Services (HHS) jointly released the Dietary Guidelines for U.S. Residents (2020–2025). Through comparative analysis, it is found that many aspects of the dietary guidelines of China and the United States are similar, such as both advocating more vegetables, fruits, and whole grains, appropriate intake of dairy and protein foods, and limiting the consumption of added sugar, sodium, and alcohol. First, compared with China, the United States pays more attention to individual variability and gives more specific recommendations for different groups of people in terms of energy intake levels and exercise, so that residents can tailor their dietary patterns according to their needs. Second, the incidence of diet-related chronic diseases, such as obesity, cardiovascular disease, type 2 diabetes, liver disease, and certain types of cancer, continues to rise among U.S. residents and is one of the major public health problems in the United States. As a result, the U.S. Dietary Guidelines focus on nutrient-dense foods and beverages from a variety of categories and keep energy intake within appropriate ranges. Our daily food intake is limited, and choosing these nutrient-dense foods is more likely to fulfill the daily nutrient requirements of the human body and avoid excessive intake of restrictive elements. Third, the Chinese Dietary Guidelines give a general recommendation, while the United States has refined and made more specific suggestions so that people of different ages can carry out the right amount of exercise according to their physical characteristics. Fourth, the quantity units used in China and the United States are different. China uses grams as a unit of quantity, while the United States uses cup equivalents and ounce equivalents as the unit, and these two units are explained in the notes through examples of some common food products, which are more operable. Fifth, due to differences in dietary habits and eating patterns, the U.S. recommends a higher intake of vegetables, fruits, and dairy than China, while the U.S. recommends a lower intake of cereals than China. In addition, the United States emphasizes the intake of fruits, fat-free milk, low-fat milk, and whole grains.

Along with the changing nutritional status and epidemiology of the global population, nutritional epidemiology has gradually shifted from single nutrients to dietary patterns, focusing on nutrition-related chronic diseases due to nutritional imbalances and the quality of an entire diet [9,10]. In the last few decades, statistical methods have emerged that fully use dietary information collected across populations to create dietary patterns [11]. In nutritional epidemiology studies, researchers in the United States have proposed scoring methods based on the American Dietary Guidelines for evaluating the dietary status of Americans and for evaluating dietary changes in populations over time, such as the Healthy Eating Index (HEI), the Dietary Quality Index (DQI), and the Mediterranean Diet Score (MDS) [12,13,14]. Due to the variability of Chinese dietary patterns, CNS established the Dietary Balance Index (DBI) based on the Chinese Dietary Guidelines and the Balanced Diet Pagoda in 2005, combined with the nutritional characteristics of Chinese people, and revised the DBI-16 to evaluate the dietary nutrition status of Chinese residents quickly [15,16]. The DBI comprises eight food group scores as indicators of food intake provided according to physical activity level. Overall diet quality, inadequacy, excesses, and imbalances were assessed using the lower limit score (LBS), higher limit score (HBS), and diet quality distance (DQD) [9]. Many studies have been conducted in China using the DBI to assess dietary quality, such as the overall nutritional quality of residents in Shandong Province, which has a moderate dietary imbalance (DQD score = 37.9).

In contrast, moderate dietary intake, inadequate and low dietary intake, and excessive intake coexist [17]. However, nutritional requirements differ by age and gender groups, and general adult DBIs cannot satisfy all populations. Many studies have revised DBIs for special populations such as pregnant women, puerperal women, the elderly, and children [18,19,20,21]. Based on the DBI-16, the latest revised Dietary Guidelines for Lactating Women in China (2022), and the Balanced Diet for Lactating Women (2022), this study establishes new scoring rules to evaluate the quality of diets of women in different cities and at various stages of lactation in China, and to provide data support for nutrition interventions targeting specific populations [4,22].

## 2. Materials and Methods

### 2.1. Participants

This cross-sectional study used data from Chinese Maternal Nutrition and Infant Investigation (MUAI). A total of 2532 healthy, breastfeeding mothers at different postpartum stages were recruited in June 2018 from six cities (Tianjin, Lanzhou of Gansu, Changchun of Jilin, Guangzhou of Guangdong, Shanghai, and Chengdu of Sichuan) from central, northwestern, northeastern, southern eastern and southwestern districts around China. In this study, 639 mothers in the first month postpartum (colostrum), 562 in the second to third month (early mature milk), and 1331 in days 200–240 postpartum (late mature milk) were investigated, with basic information and food frequency questionnaires carried out in the last month. The inclusion criteria of the study subjects were as follows: lactating mothers aged 18–40 years who had lived in the region for more than two years, had a singleton, and a full-time delivery; and lactating mothers who had no significant dietary preferences and were breastfeeding until six months and more. Participants with hypertension, diabetes, other metabolic diseases, acute and chronic infectious diseases, and those who have taken drugs that affect nutrient metabolism were excluded. The MUAI study was registered at the Chinese Clinical Trial Center (ChiCTR1800015387). All procedures were approved by the Ethics Committee (Approval No. XHCE-C-2017-064). Finally, all participants in this investigation signed informed consent forms, and the privacy and confidentiality of the personal information of the mothers participating in the survey will be protected.

### 2.2. Measurements

Food frequency investigation: the investigators interviewed the participating mothers and their families to obtain basic information about the lactating mothers and their newborns, including the age, pregnancy, delivery, and prenatal weight of the lactating mothers, and the birth weight, sex, and length of the newborns.

The investigators inquired about the quantity and frequency of food consumed by the enrolled lactating mothers in the past month through face-to-face interviews, mainly concerning the type and quantity of food intake and the frequency and average single serving size of each type. The dietary frequency questionnaire covered 118 food items: cereals, potatoes, fruits, vegetables, meat, dairy, eggs, fish and shrimp, soybeans and nuts, dietary supplements and snack desserts, alcoholic beverages, and condiments. During the survey, standard food volume reference pictures, bowls, spoons, and other tableware were used to determine the single serving size of each food type.

We adjusted the DBI-16 for lactating women. The DBI-16 is composed of 8 food group-level indicators. The selection of indicators and the setting of scores reflect the core elements of the Chinese Dietary Guidelines and the Chinese Balanced Diet Pita (2016), including cereal foods, vegetables and fruits, dairy and soybeans, animal foods, alcohol, salt, edible oils, food groups, and drinking water, but are applicable to the dietary assessment of adults. This study was adjusted according to the revised dietary guidelines and dietary pagoda for lactating women in China by CNS in 2022, and the detailed scoring criteria are shown in Table 1 and Table 2 [4]. The revised DBI in this study consists of six components: C1—cereals; C2—vegetable and fruit; C3—dairy, soybean, and nut; C4—animal food; C5—empty energy food, and C6—condiments, with a range of scores for each component and indicator, a value of “0” for each indicator when the recommended amount is reached, and negative scores indicating inadequate food intake and positive scores indicating excessive food intake. For foods emphasized as “eat more” or “eat more often” in the dietary guidelines, the value of these indicators was negative. The value of these indicators was negative for foods that are emphasized as “eat less” in the guidelines. For foods that are emphasized in the guidelines as “less”, the focus was on the degree of overconsumption, and the values of these indicators were positive; for foods that are emphasized in the guidelines as “moderate”, the evaluation should reflect both underconsumption and overconsumption and the values of these indicators are both positive and negative. This study did not include data on drinking water. Both positive and negative scores were used to assess intake levels of cereals (scores −12 to 12) and animal foods (scores −12 to 8), which are recommended to be consumed in “appropriate” amounts, across the six indicators. Negative scores were used to assess inadequate intake levels of vegetables and fruits (scores −6 to 0), dairy, soybean, and nut (scores −12 to 0), and the guidelines recommend that women in the puerperium should consume “adequate” or “sufficient” amounts. Positive scores (scores 0 to 12) were used to assess empty energy foods and condiments, and the guidelines recommend that lactating women consume “little” or “none” of these foods. Considering the difference in nutritional requirements according to energy consumption, lactating mothers are considered to be lightly physically active. The Chinese Dietary Reference Intakes (DRIs) recommend 2300 kcal daily energy intake for lactating mothers; therefore, different food recommendations are given concerning the 2200 kcal energy level in the DBI-16 [23].

Calculation of the scores was still based on the calculation method of DBI-16 scores, which includes total score (TS), high bound score (HBS), low bound score (LBS), and diet quality distance (DQD). The scores were evaluated: 0 was considered “excellent”, and less than 20% of the total score was considered “almost no problem”. The following scores were considered poor levels: 20% to 40% of the total score was low level, 40% to 60% of the total score was moderate level, and above 60% was high level. Therefore, HBS was the absolute value of the sum of positive scores of all indicators reflecting the degree of excessive dietary intake, for which the range of scores is 0~44. A score of 0 means no excessive intake, which indicates “excellent”; 1~9 means “almost no problem”, which indicates “good”; 10~18 means low excessive intake, which indicates “mild poor”; 19~27 means moderate excessive intake, which indicates “moderately poor”, and more than 27 means high excessive intake which indicates “severely poor”. LBS was the absolute value of adding up the negative scores of all indicators. Since there is no water intake data, the score range is 0~48 and is represented in the paper as above. A score of 0 means no inadequate intake, 1~10 means “almost no problem”, 11~19 means low inadequate intake, 20~28 means moderate inadequate intake, and more than 28 means inadequate high intake. DQD is the absolute value of each index score that is added to reflect the dietary balance problems. A score of 0 means there is neither inadequate nor excessive intake. The range of scores is 0~72, and scores of 1~14 mean “almost no problem”, 15~29 mean low dietary imbalance, 30~43 mean moderate dietary imbalance, and scores over 43 mean high dietary imbalance.

### 2.3. Statistical Analysis

Statistical analyses were performed using SPSS software 23.0 (SPSS Inc. Chicago, IL, USA) and Microsoft Office Excel (2021, Redmond, WA, USA). Demographic characteristics of lactating mothers were described as mean ± standard deviation for continuous variables and number (percentage) for categorical variables. As the dietary data conformed to a normal distribution, continuous variables were expressed as mean ± standard deviation. Differences in dietary data between urban and lactation stages were derived using ANOVA (continuous variables) and Chi-squared tests (categorical variables).

## 3. Results

### 3.1. Characteristics of the Participants

The basic characteristics of the postpartum mothers and newborns who participated in this investigation are shown in Table 3. A total of 2532 mothers in three lactation stages participated in this investigation, 639 in 0–30 days postpartum, 562 in 40–90 days postpartum, and 1331 in 200–240 days postpartum. The age range of the lactating mothers was 19–44 years, with a mean age of 29.75 years, and 89.8% were younger than 35 years. The mean pre-pregnancy and predelivery BMI values were 21.01 kg/m^2^ and 26.41 kg/m^2^, respectively. The mean gestational age of the newborns was 39.29 weeks, and the mean length and weight were 49.96 cm and 3318.79 g. All newborns had Apgar scores greater than 8, and 1796 were male, accounting for 51.3%.

### 3.2. Distribution of DBI Components in Food Intake Scores of Lactating Women

The scores for the DBI for lactation (DBI-L) components and the percentage of participants for each score are shown in Table 4. Overall, except for empty energy foods and condiments, the percentage of participants who met the recommended dietary intake ranged from 7.0% to 60.3% (score = 0). Overall, 76.0%, 64.4%, and 29.1% of mothers had excessive intakes of cereals, meat, and eggs (score > 0), and 79.1%, 79.1%, 86.7%, 39.7%, and 69.4% had inadequate intakes of vegetables, fruits, dairy products, soybean and nuts, and fish (score < 0). For empty energy foods, 98% and 98.3% of mothers met the recommended intake of oil and alcoholic beverages (score = 0). A total of 98.7% of mothers met the recommended intake of added sugar (score = 0), but 94.2% of mothers had excessive salt intake (score > 0).

### 3.3. Distribution of Diet Quality Indicators by DBI for Lactating Mothers

The mean ± SD values of overall HBS, LBS, and DQD were 10.99 ± 5.77, 14.50 ± 7.43 and 25.47 ± 7.8, respectively. The distribution of these indicators is shown in Table 5. The sum percentages of HBS, LBS, and DQD in the “excellent” and “good” levels were 42.2%, 33.8%, and 7.8%, respectively. The sum percentages of HBS, LBS, and DQD in the “poor” level were 57.8%, 66.2%, and 92.2%, respectively. The results showed that more than half of the lactating mothers had problems with excessive and inadequate dietary intake, and most had imbalanced dietary intake.

As shown in Table 6, the differences in HBS, LBS, and DQD were significant across lactation stages and cities (*p* < 0.05). For the lactation stage, the mean values of HBS, LBS, and DQD at 30 days postpartum were 9.45, 13.38, and 22.83, respectively, lower than those at 40–90 days and 200–240 days postpartum. In terms of cities, Changchun had a lower HBS mean value of 9.06 than other cities and a significantly higher LBS and DQD mean value of 21.89 and 30.95 than other cities. The mean values of LBS and DQD in Shanghai were 10.31 and 21.01, significantly lower than in other cities (*p* < 0.05).

### 3.4. Dietary Assessment of Lactating Mothers in Different Cities

The dietary intake of lactating mothers in different cities is shown in Table 7. The maternal cereal intake in all six cities was significantly higher than the recommended intake. Guangzhou and Lanzhou had a higher average daily intake of 565.68 g and 547.66 g than other cities (*p* < 0.05). The average daily intake of vegetables for lactating mothers in Tianjin was 425.13 g, and the average intake of fruits in Shanghai was 230.31 g, significantly higher than in other cities. Regarding dairy products, the average daily intake was generally lower than the recommended value, with Changchun at 90.47 g, significantly lower than other cities. Regarding animal food, the average daily meat intake was excessive, while fish and shrimp intake was generally inadequate. The average daily meat intake in Guangzhou was higher than that in other cities, and the intake of fish and shrimp in Shanghai was higher than in other cities.

**Figure 1:**
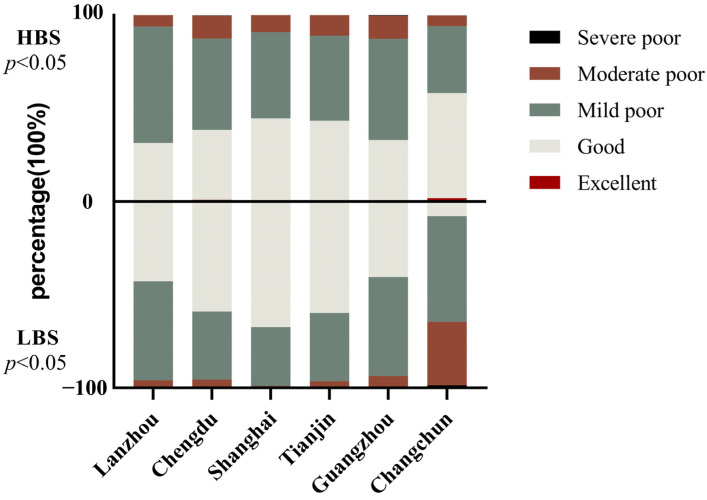
Distribution of HBS and LBS among different cities. HBS: higher bound score of Dietary Balance Index for urban Chinese lactating women, excessive intake indicator; LBS: lower bound score of Dietary Balance Index for urban Chinese lactating women, inadequate intake indicator. Score range of HBS is 0–44; excellent: no problem, score = 0; good: almost no problem, 1–9; mild poor: low level, 10–18; moderate poor: moderate level, 19–27; severe poor, high level: 27–44. The score range of LBS is 0–48; no problem: 0; almost no problem: 1–10; low level: 11–19; moderate level: 20–28; high level: 29–48. Here, *p* values (2-sided) were derived using Chi-test (categorical variables), and *p* < 0.05 indicates a significant difference.

The distribution of HBS and LBS among different cities is shown in Figure 1. We found that excessive and inadequate dietary intake coexisted across cities. Shanghai had the highest proportion of LBS and HBS at “good” levels, and the lowest proportion of lactating mothers at poor levels. Lanzhou had a higher proportion of lactating mothers with excessive intake; Changchun had a higher proportion of inadequate intake than other cities.

### 3.5. The Distribution of the Diet Quality Distance of Lactating Mothers

**Figure 2:**
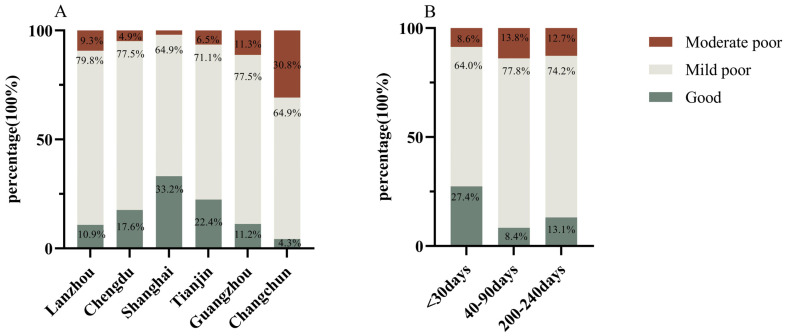
Diet quality distance (DQD) distribution among different cities (**A**) and lactation stages (**B**). DQD: diet quality distance of Dietary Balance Index for urban, Chinese lactating women, overall imbalanced food intake indicator. The score range of DQD is 0–72: good means almost no problem, score range of 1–14; mild poor means low level, score range 15–29; moderate poor means moderate level, score range of 30–43. A period of <30 days, 40–90 days, 200–240 days, the mean period of postpartum. Here, *p* values (2-sided) were derived using Chi-test (categorical variables), and *p* < 0.05 indicates a significant difference.

The distribution of the diet quality distance of lactating mothers across lactation stages (A) and cities (B) is shown in Figure 2. Most lactating mothers had DQD at a mild poor level, and no mothers were at excellent or severe poor levels.

Regarding the DQD level of breastfeeding mothers in different cities, the percentage of mothers with “mild poor” in Lanzhou was 79.8%, higher than in other cities. The percentage of mothers with “good” in Shanghai was 33.2%, higher than in other cities; the percentage of mothers with “moderately poor” was 1.9%, lower than in other cities. The percentage of “moderately poor” mothers in Changchun was 30.8%, higher than in other cities; the percentage of “good” mothers was 4.3%, lower than in other cities (*p* < 0.05).

Regarding different lactation periods, 27.4% of mothers had good diets in the first month postpartum, a higher percentage than the other two periods, and 8.6% of mothers had moderately poor diets, lower than the other two lactation stages. In the 2 to 3 months postpartum, 77.8% and 13.8% of mothers had mild, moderately poor diets, with a higher percentage than the other two lactation periods, respectively, and 8.4% had good diets, with a lower percentage than the other two lactation periods (*p* < 0.05).

## 4. Discussion

Although there have been some reports on maternal dietary nutrition in China, this study was the first to assess the dietary quality of lactating mothers according to the dietary index method adjusted from the Chinese Dietary Guidelines for Lactating Women (2022). This study covered different urban areas and different stages of lactation in China. This study provided a more comprehensive picture of the nutritional status of Chinese women’s diets throughout the lactation period, filled a gap in assessing lactating women’s diets using an oriental dietary pattern, and provided data to support targeted nutritional interventions for lactating women.

Most studies or reviews of nutritional epidemiology classify statistical methods for dietary pattern analysis into researcher-driven and data-driven methods [10,24,25,26,27]. Investigator-driven methods, also known as priori methods, are defined as dietary guidelines consistent with current nutritional knowledge or dietary recommendations; they affect health as dietary patterns that score foods or nutrients consumed according to a quality score and aggregate the results to produce dietary quality scores such as dietary scores and dietary indexes [27,28,29]. For example, several studies on the relationship between diet quality scores and health have shown that scores on the HEI, Alternative Healthy Eating Index (AHEI), Dietary Alternative Mediterranean Diet (DASH), and DBI are correlated with the risk of death from cardiovascular disease, cancer, all-cause mortality, and postnatal obesity [19,30,31,32,33]. Data-driven methods, also known as posteriori methods, refer to dietary intake patterns derived from available data collected through food frequency questionnaires, 24 h recall questionnaires, or dietary records, most often using principal component analysis (PCA) and exploratory factor analysis (EFA) methods [34]. Since investigator-driven methods are hypothesis-oriented methods that neither reflect overall dietary patterns nor consider the structure of nutrient correlations, and data-driven methods do not consider any a priori expertise on health outcomes, hybrid methods combining the above two types of methods are also often used in nutritional epidemiology studies [10,35]. In this study, the Chinese Dietary Balance Index was assessed using an investigator-driven method that has been validated in several studies as a valid indicator of dietary quality for assessing populations through excess and inadequate dietary intake [8,19,36]. The Mediterranean diet score (MDS) and healthy eating index (HEI), which are more widely used abroad, apply dietary patterns that are certainly different from the traditional dietary patterns in China [12,37]. Although the China healthy diet index (CHDI) can reflect the consistency of individual diets with guideline recommendations, it cannot give a two-way assessment [38].

The DBI-2016 applies to normal adults; this study was revised to meet the needs of the lactating female population according to the Chinese Dietary Guidelines for Lactating Women (2022) recommendations. Considering the mainly sedentary and lying down rest during the “Zuo Yue Zi” of lactating women and the dietary control for weight restoration, our study estimated the energy level to be 2200 Kcal/d according to the DBI-16. In addition, the guidelines (2022 edition) recommend an increase in the intake of animal foods abundant in high-quality protein and vitamin A during lactation and an increase in milk intake for calcium supplementation. Therefore, this study adjusted the scoring rules for the relevant components in the DBI [4]. The guidelines emphasize that lactating women should prohibit alcohol intake and limit sugar and oil intake, and we also adjusted these items. As drinking data were not collected in this study, the range of values for LBS and DQD were adjusted accordingly. However, considering the variability of national dietary preferences, the DBI is not universal, and comparison with dietary data from other countries is not recommended. We suggest that the corresponding dietary balance indexes be developed according to the dietary guidelines of different countries.

The study results showed that the dietary intake of lactating mothers was imbalanced, and excessive and insufficient intake issues coexisted. In general, 72.3% of lactating mothers had a mild dietary imbalance, with inadequate intake being the primary cause of dietary imbalance. Consistent with previous studies, lactating mothers had a significantly inadequate intake of vegetables, fruits, dairy, soy nuts, and fish and shrimp, which are good sources of protein, minerals, and unsaturated fatty acids and should be taken into account for targeted supplementation during lactation. The study also show a significant excessive intake of cereals, eggs, meat, and salt in lactating mothers, which is consistent with the results of the study in Guangzhou [39,40,41]. Several studies in Xiamen, Fuzhou, and Hubei Provence showed that postpartum women were abundant in meat, poultry, and egg consumption but inadequate in vegetables, fruits, and dairy products, similar to our study [6,42,43]. A study of postpartum women in central China showed that postpartum women consumed more cereals and animal foods. At the same time, the consumption of fruits, vegetables, and dairy products did not reach the recommended values, which is consistent with our results [44]. This result may be related to the traditional dietary concepts in China, as surveys have shown that most people believe that raw and cold foods, such as vegetables, fruits, dairy, and soybeans, should be abstained from postpartum diets, which would cause multiple vitamin and mineral deficiencies in postpartum women [45]. In addition, excessive meat intake from livestock and poultry leads to a severe excess of dietary fat intake, and studies have shown that the rate of obesity in women after childbirth is substantially higher than before pregnancy [43]. Similar results have been reported in other countries, and studies have reported that lactating mothers’ consumption of vegetables and fruits in Spain and the USA did not meet their national recommendations [46,47].

In terms of dietary quality of lactating mothers at different stages of lactation, this study found that the values of HBS, LBS, and DQD of mothers at one month postpartum were less than those of other mothers at 2–3 months postpartum and more than six months postpartum (*p* < 0.05). In addition, the highest percentage of lactating mothers with good dietary quality was 27.4%, and the lowest percentage of moderate dietary quality imbalance was 8.6% at one month postpartum. We believe that the dietary intake of lactating mothers in the first month after delivery is more balanced than in other stages of lactation, and some studies have shown that the average daily intake of eggs, fish, and meat in the first month after delivery is higher than that of women in the puerperium [6,43]. Some studies suggest that this result is due to reduced attention to diet of lactating mothers after the “Zuo Yue Zi” period [6,43].

The geographical area of China is extensive, and different geographic environments have created widely varying dietary habits. This study’s HBS, LBS, and DQD values differed significantly between cities (*p* < 0.05). Among them, Changchun had a more pronounced dietary deficiency problem and the most unbalanced diet. Guangzhou had a more pronounced dietary excess problem, and Shanghai had a more balanced diet. In this study, fish and shrimp consumption for coastal Shanghai and Guangzhou lactating mothers was higher than that for lactating mothers from inland cities such as Changchun Lanzhou Chengdu. The American Dietary Guidelines state that an increased frequency in the consumption of seafood and other aquatic products by lactating mothers can significantly increase the level of DHA in breast milk and affect fetal neurological and visual development [48]. Consumption of meat, eggs, fruits, and milk by lactating mothers was higher in economically developed Shanghai and Guangzhou than in less economically developed Changchun and Lanzhou. Cereal consumption by lactating mothers in less economically developed areas such as Lanzhou and Changchun was significantly higher than in other cities. Studies have shown that economic income is an essential factor influencing the dietary structure of Chinese residents [49]. With economic development, the traditional dietary pattern of plant-based foods characterized by high carbohydrates, high dietary fiber, and low fat has transitioned to a dietary structure dominated by animal-based foods, and this phenomenon is more evident among cities of different economic levels [50].

## 5. Conclusions

In summary, the quality of lactating women’s diets is important to maternal health and offspring development. Overall, dietary excess and inadequate intake of lactating mothers in China coexist, and the dietary quality of lactating mothers varies across lactation stages and between cities. To our knowledge, this is the first study to apply the latest version of the guideline-adjusted dietary index to assess overall maternal diet quality in China. The results suggest that maternal diets are unbalanced and dominated by inadequate dietary intake. We believe this study assesses the validity and reliability of the adjusted DBI, establishes a comparison of DBIs across countries, and further explores the relationship between dietary nutrients and breast milk composition.

We believe that the country should develop appropriate policies to implement targeted nutritional interventions at different stages of lactation and in different regions, especially in the less economically developed northeast and northwest regions.

## Figures and Tables

**Table 1:** Food groups of the adjusted Dietary Balance Index for lactating women.

Components	Representative Foods	Score Ranges
C1—cereal	Rice, pasta, coarse grains, potatoes, and mixed beans	(−12)–12
C2—vegetable and fruit	Dark vegetables	(−6)–0
	light vegetables	
	Fruits	(−6)–0
C3—dairy, soybean, and nut	Dairy	(−6)–0
	Soybean and nut	(−6)–0
C4—animal food	Meat and poultry (animal offal)	(−4)–4
	Fish and shrimp	(−4)–0
	Eggs	(−4)–4
C5—empty energy food	Cooking oil	0–6
	Alcoholic beverage	0–6
C6—condiments	Added sugar	0–6
	Salt	0–6

**Table 2:** Components of the adjusted Dietary Balance Index for lactating women.

Components	Groups	Range	Guidelines	Scoring Definition
C1—cereal ^a^	Cereal	(−12)−12	Cereal 225–275 g, potato 75 g	0 g = −12; 275–325 g = 0; >600 g = 12; score increases 1 with intake amount decrease 25 g.
C2—vegetable and fruit	Vegetable	(−6)−0	400–500 g	≥450 g = 0; 360–449 g = (−1); score decrease 1 with intake amount decrease 90 g; 0 g = (−6).
	Fruit	(−6)−0	200–350 g	≥300 g = 0; 240–299 g = (−1); score decrease 1 with intake amount decrease 60 g; 0 g = (−6).
C3—dairy, soybean, and nut	Dairy	(−6)−0	300–500	≥400 g = 0; Score decreased 1 with intake amount decreased by 80 g; 0 g = −6
	soybean and nut	(−6)−0	Soybean 25 g, nut 10 g	≥35 g = 0; 28–34 = −1; score decrease 1 with intake amount decrease 7 g; 0 g = (−6).
C4—animal food	Meat and poultry	(−4)−4	50–75 g	0 g = (−4); 1–25 g = (−3); 26–50 g = (−2); 51–75 g = (−1); 76–95 g = 0; 96–120 g = 1; 121–145 g = 2; 146–170 g = 3; >170 g = 4.
	Fish and shrimp	(−4)−0	75–100 g	<15 g = (−4); 15–34 g =(−3); 35–54 g = (−2); 55–74 g = (−1); ≥75 g = 0.
	Eggs	(−4)−4	50 g	0 g = −4; 1–15 g = −3; 16–30 g = −2; 31–45 g = −1; 46–55 g = 0;56–70 g = 1; 71–85 g = 2; 86–100 g= 3; >100 g =4
C5—empty energy food	Cooking oil	0–6	25 g	≤25 g = 0; 26–30 g = 1; >50 g = 6
	Alcoholic beverage ^b^	0–6	0	0 g = 0; 1–10 g = 1; score increases 1 with intake amount increase 10 g; >50 g = 6.
C6—condiments	Added sugar	0–6	25 g	≤25 g = 0; 26–30 g = 1; score increase 1 with intake amount increase 5 g; >50 g = 6.
	Salt	0–6	<5 g	<5 g = 0; 5–6 g = 1; 7–8 g = 2; score increase 1 with intake amount increase 2 g; >15 g = 6.
C7—food variety	Food variety	(−12)−0	12	≥12 kinds of food (soybean is 5 g) = 0; Score decreased by 1 with food variety decreased by 1.

The DRIs recommend a daily energy intake of 2300 kcal for lactating women who are at a light physical activity level. ^a^ Cereal includes rice, wheat, dried legumes (exclude soybean), and tubers. Intake amount means fresh amount. ^b^ 25 g alcohol = 750 mL beer or 250 mL wine or 75 g 38° liquor 38° or 50 g liquor > 38°.

**Table 3:** Basic information of participating mothers and newborns.

Characteristics (*n* = 2532)	*n*	Percentage (%)
Age (years)		
Mean ± SD	29.75 ± 3.55	
19–34	2275	89.8
35–44	257	10.2
Gestational age (weeks)		
Mean ± SD	39.29 ± 1.75	
Pre-pregnancy BMI (kg/m^2^)		
Mean ± SD	21.01 ± 2.88	
Predelivery BMI (kg/m^2^)		
Mean ± SD	26.41 ± 3.19	
Primary delivery	1790	70.7
Vaginal delivery	1653	65.3
Lactation stage		
<30 days	639	25.2
40–90 days	562	22.2
200–240 days	1331	52.6
Offspring gender		
Male	1296	51.3
Offspring weight (g)		
Mean ± SD	3318.79 ± 441.40	
Offspring length (cm)		
Mean ± SD	49.96 ± 2.20	

SD: standard deviation; BMI: body mass index.

**Table 4:** Distribution of DBI-L components and percentage of participants’ scores (%).

DBI-L ^a^ Components	Mean	−12~	−10~	−8~	−6~	−4~	−2~	0	1~	3~	5~	7~	9~	11~
Cereal	5.63	0.6	0.5	1.1	2.9	4	5.9	9	7.7	9.7	9.8	8.6	7.5	32.7
Vegetable	−2.48				10.8	43.0	25.3	20.9						
Fruit	−2.97				26.8	32.7	19.6	20.9						
Dairy	−3.26				34.8	20.1	31.8	13.3						
Soybean and nut	−1.73				21.5	9.8	8.4	60.3						
Meat and poultry	1.29					9.0	19.6	7.0	22.6	41.8				
Fish and shrimp	−2.04					44.0	25.4	30.6						
Egg	0.02					17.0	12.5	41.4	7.7	21.4				
Cooking oil	0.04							98.3	1.2	0.4	0.2			
Alcoholic beverage	0.08							98.0	0.7	0.6	0.8			
Added sugar	0.09							97.9	0.4	0.2	1.4			
Salt	1.85							5.8	77.6	9.1	7.5			

^a^ The score range was different for each food subgroup: cereal (−12~12), vegetables (−6~0), fruits (−6~0), dairy (−6~0), soybean (−6~0), meat and poultry (−4~4), fish and shrimp (−4~0), eggs (−4~4), cooking oil (0–6), alcoholic beverage (0–6), added sugar (0–6), salt (0–6).

**Table 5:** Distribution of DBI-L indicators among lactating mothers.

Indicator	Mean ± SD	Excellent ^#^	Good	Mild Poor	Moderate Poor	Severe Poor
HBS ^a^	10.99 ± 5.77	16 (0.6)	1054 (41.6)	1225 (48.4)	235 (9.3)	2 (0.1)
LBS ^b^	14.50 ± 7.43	4 (0.2)	850 (33.6)	1060 (41.9)	516 (20.4)	102 (4.0)
DQD ^c^	25.47 ± 7.87	0 (0.0)	198 (7.8)	1581 (62.4)	705 (27.8)	48 (1.9)

^a^ HBS: higher bound score of Dietary Balance Index for urban Chinese lactating women, excessive intake indicator; ^b^ LBS: lower bound score of Dietary Balance Index for urban Chinese lactating women, inadequate intake indicator; ^c^ DQD: diet quality distance of Dietary Balance Index for urban Chinese lactating women, overall imbalanced food intake indicator. The score range of HBS is 0–44; ^#^ excellent: no problem, score = 0; good: almost no problem, 1–9; mild poor: low level, 10–18; moderate poor: moderate level, 19–27; severe poor, high level: 28–44. The score range of LBS is 0–48; no problem: 0; almost no problem: 1–10; low level: 11–19; moderate level: 20–28; high level: 29–48. The score range of DQD is 0–72; no problem: 0; almost no problem: 1–14; low level: 15–29; moderate level: 30–43; high level: 44–72.

**Table 6:** Distribution of DBI indicators according to different lactation stages and cities.

	HBS		LBS		DQD	
	Mean ± SD	*p*	Mean ± SD	*p*	Mean ± SD	*p*
Postpartum						
<30 days	9.45 ± 5.63 ^b^	<0.05	13.38 ± 8.32 ^b^	<0.05	22.83 ± 8.64 ^b^	<0.05
40–90 days	11.40 ± 5.57 ^a^		15.43 ± 6.64 ^a^		26.83 ± 6.98 ^a^	
200–240 days	11.56 ± 5.8 ^a^		14.61 ± 7.26 ^a^		26.17 ± 7.54 ^a^	
Cities						
Lanzhou	11.71 ± 4.89 ^ab^	<0.05	13.88 ± 5.92 ^b^	<0.05	25.59 ± 6.62 ^b^	<0.05
Chengdu	11.62 ± 6.14 ^a^		12.19 ± 6.22 ^c^		23.80 ± 6.88 ^c^	
Shanghai	10.70 ± 5.81 ^b^		10.31 ± 5.35 ^d^		21.01 ± 6.85 ^d^	
Tianjin	10.90 ± 6.09 ^b^		11.89 ± 6.51 ^c^		22.79 ± 7.09 ^c^	
Guangzhou	12.47 ± 5.55 ^a^		14.01 ± 6.61 ^b^		26.48 ± 7.29 ^b^	
Changchun	9.06 ± 5.48 ^c^		21.89 ± 6.72 ^a^		30.95 ± 7.82 ^a^	

Data are expressed as means ± standard deviations for continuous variables. Here, *p* values (2-sided) were derived using ANOVA (continuous variables), and *p* < 0.05 indicates a significant difference. ^abcd^ Values within a column with different superscript letters were significantly different (*p* < 0.05).

**Table 7:** Distribution of dietary intake of lactating mothers in different cities.

	RNI (g/day)	Lanzhou (*n* = 397)	Chengdu *(n* = 409)	Shanghai (*n* = 365)	Tianjin *(n* = 402)	Guangzhou (*n* = 427)	Changchun (*n* = 532)	*p*
Cereal	300–350	547.66 ± 192.27 ^a^	526.74 ± 300.70 ^b^	481.17 ± 202.26 ^c^	499.38 ± 264.15 ^bc^	565.68 ± 311.41 ^a^	421.44 ± 197.75 ^c^	<0.05
Vegetable	400–500	253.11 ± 132.43 ^c^	382.75 ± 286.24 ^b^	348.07 ± 181.89 ^b^	425.13 ± 210.53 ^a^	287.05 ± 232.50 ^c^	179.15 ± 132.80 ^d^	<0.05
Fruit	200–350	165.61 ± 140.60 ^b^	229.88 ± 257.75 ^a^	230.31 ± 167.33 ^a^	172.37 ± 159.48 ^b^	143.68 ± 144.05 ^b^	146.50 ± 176.83 ^b^	<0.05
Dairy	300–500	176.58 ± 157.93 ^c^	250.36 ± 177.44 ^ab^	255.93 ± 189.17 ^a^	223.68 ± 177.60 ^b^	220.44 ± 209.88 ^b^	90.47 ± 150.08 ^d^	<0.05
Soybean and nut	35	99.59 ± 91.53 ^b^	83.3 ± 97.216 ^bc^	78.7 ± 94.33 ^c^	124.73 ± 147.20 ^a^	73.07 ± 105.88 ^c^	46.84 ± 87.10 ^d^	<0.05
Meat and poultry	50–75	106.70 ± 85.58 ^c^	177.98 ± 178.11 ^b^	169.53 ± 125.06 ^b^	115.11 ± 94.15 ^c^	249.53 ± 172.45 ^a^	116.73 ± 108.10 ^c^	<0.05
Fish and shrimp	75–100	56.21 ± 76.93 ^c^	59.28 ± 72.26 ^c^	107.75 ± 103.81 ^a^	67.51 ± 76.30 ^c^	84.18 ± 109.41 ^b^	37.15 ± 77.06 ^d^	<0.05
Egg	50	48.46 ± 29.04 ^c^	60.75 ± 56.47 ^ab^	58.24 ± 35.01 ^b^	68.99 ± 47.48 ^a^	47.53 ± 66.53 ^c^	65.99 ± 70.08 ^ab^	<0.05

RNI = recommended nutrient intake; data are expressed as means ± standard deviations for continuous variables. Here, *p* values (2-sided) were derived using ANOVA (continuous variables), and *p* < 0.05 indicates a significant difference. ^abcd^ values within a row with different superscript letters were significantly different (*p* < 0.05).

## Data Availability

The data are available in a publicly accessible repository.
